# Lewis A Glycans Are Present on Proteins Involved in Cell Wall Biosynthesis and Appear Evolutionarily Conserved Among Natural *Arabidopsis thaliana* Accessions

**DOI:** 10.3389/fpls.2021.630891

**Published:** 2021-03-11

**Authors:** Gernot Beihammer, Daniel Maresch, Friedrich Altmann, Els J. M. Van Damme, Richard Strasser

**Affiliations:** ^1^Department of Applied Genetics and Cell Biology, Institute of Plant Biotechnology and Cell Biology, University of Natural Resources and Life Sciences, Vienna, Austria; ^2^Division of Biochemistry, Department of Chemistry, University of Natural Resources and Life Sciences, Vienna, Austria; ^3^Laboratory of Biochemistry and Glycobiology, Department of Biotechnology, Ghent University, Ghent, Belgium

**Keywords:** glycosylation, N-glycans, carbohydrate epitope, glycoproteomics, posttranslational modification

## Abstract

N-glycosylation is a highly abundant protein modification present in all domains of life. Terminal sugar residues on complex-type N-glycans mediate various crucial biological processes in mammals such as cell-cell recognition or protein-ligand interactions. In plants, the Lewis A trisaccharide constitutes the only known outer-chain elongation of complex N-glycans. Lewis A containing complex N-glycans appear evolutionary conserved, having been identified in all plant species analyzed so far. Despite their ubiquitous occurrence, the biological function of this complex N-glycan modification is currently unknown. Here, we report the identification of Lewis A bearing glycoproteins from three different plant species: *Arabidopsis thaliana, Nicotiana benthamiana*, and *Oryza sativa*. Affinity purification via the JIM84 antibody, directed against Lewis A structures on complex plant N-glycans, was used to enrich Lewis A bearing glycoproteins, which were subsequently identified via nano-LC-MS. Selected identified proteins were recombinantly expressed and the presence of Lewis A confirmed via immunoblotting and site-specific N-glycan analysis. While the proteins identified in *O. sativa* are associated with diverse functions, proteins from *A. thaliana* and *N. benthamiana* are mainly involved in cell wall biosynthesis. However, a Lewis A-deficient mutant line of *A. thaliana* showed no change in abundance of cell wall constituents such as cellulose or lignin. Furthermore, we investigated the presence of Lewis A structures in selected accessions from the 1001 genome database containing amino acid variations in the enzymes required for Lewis A biosynthesis. Besides one relict line showing no detectable levels of Lewis A, the modification was present in all other tested accessions. The data provided here comprises the so far first attempt at identifying Lewis A bearing glycoproteins across different species and will help to shed more light on the role of Lewis A structures in plants.

## Introduction

N-glycosylation is an abundant protein modification present in all domains of life. N-glycosylation is initiated by the transfer of a preassembled oligosaccharide to certain asparagine residues of nascent polypeptide chains either co-translationally as they enter the lumen of the endoplasmic reticulum (ER), or post-translationally. The attached oligosaccharide is subsequently processed in the ER and Golgi, resulting in a wide variety of structures which can differ greatly between genera (Wang et al., 2017). The initial steps of N-glycan processing are evolutionarily conserved between plants and animals, i.e., cleavage of three glucoses and one mannose in the ER, and further removal of mannoses and attachment of N-acetylglucosamine (GlcNAc) in the *cis*-Golgi (Strasser, 2016). Differentiation of N-glycans between plants and animals starts in the medial-Golgi. Common processing steps include the cleavage of two more mannoses and the attachment of a second GlcNAc residue. Plants additionally add a xylose in β1,2-linkage to the N-glycan and a fucose residue is attached in α1,3-linkage to the core GlcNAc. The overall variety of N-glycan structures in plants is limited compared to animals. While animals produce biantennary or further branched N-glycans with additional sugars such as sialic acid, the only known outer chain elongation of complex N-glycans are Lewis A structures (Fitchette-Lainé et al., 1997). This trisaccharide consists of terminal GlcNAc, to which β1,3-galactose and α1,4-fucose are attached [Fucα1,4(Galβ1,3)GlcNAc-R]. The biosynthesis of Lewis A takes place in the *trans*-Golgi and is a sequential process, in which first the β1,3-galactosyltransferase (GALT1) attaches a galactose residue (Strasser et al., 2007) and afterwards the α1,4-fucosyltransferase (FUT13) attaches a fucose (Wilson, 2001, Léonard et al., 2002). In the model plant *A. thaliana*, Lewis A bearing complex N-glycans show organ-specific expression, being absent in leaves but present in stems and siliques. However, also in these plant organs they constitute only a minor fraction of the total N-glycan content (Strasser et al., 2007) indicating that only few proteins are modified with this trisaccharide. Overall, this modification appears to be evolutionarily conserved, having been identified in mosses such as *Physcomitrella patens* (Koprivova et al., 2003), a wide variety of foodstuff (Wilson et al., 2001, Okada et al., 2017), and on diverse water plants (Maeda et al., 2016). Despite its widespread occurrence, the biological role of Lewis A structures has not been identified. Lewis A-deficient plants of *A. thaliana* lack an obvious phenotype (Strasser et al., 2007; Strasser, 2016) and, so far, the knowledge about proteins bearing Lewis A is rather scarce. Two publications investigating the glycoproteome of Arabidopsis inflorescence stems (Zhang et al., 2011; Zeng et al., 2018) have identified in total four proteins decorated with Lewis A structures. Yet a study dedicated to the isolation of Lewis A bearing glycoproteins has not been published. Here, we describe the identification of glycoproteins modified with Lewis A structures from three different plant species and show that Lewis A structures represent an evolutionarily conserved modification in natural accessions of *A. thaliana*.

## Materials and Methods

### Plant Material and Growth Conditions

Seeds of ecotypes and mutants of *A. thaliana* were sterilized in 4% (v/v) NaOCl solution prior to being sown to half-strength Murashige-Skoog (MS) plates containing 1% (v/w) sucrose and 0.8% (w/v) agar. Plants were grown under long-day conditions (16 h light/ 8 h dark) at 22°C and transferred to soil 7 days after sowing. The *galt1 fut13* double knockout mutant was obtained by crossing previously described lines *galt1* (N871760) and *fut13* (N567444) (Strasser et al., 2007). Except Col-0, all described ecotypes were ordered from the Nottingham Arabidopsis Stock Centre (NASC). Wild-type and ΔXT/FT *N. benthamiana* plants (Strasser et al., 2008) were grown at 24°C at 16 h light/8 h dark for 5 weeks. Shoots of *Oryza sativa japonica* cv Nipponbare were grown for 10 days as described (Lambin et al., 2020).

### Isolation of Lewis A Bearing Glycoproteins

For isolation of Lewis A bearing glycoproteins, leaves and stems of 5-week-old *N. benthamiana*, stems and siliques of 6- and 8-week-old *A. thaliana* Col-0 plants, respectively, and shoots of 10-day-old *O. sativa* plants were used. Plant material was snap-frozen, grinded, and total proteins extracted using Radio-Immunoprecipitation assay (RIPA) buffer [50 mM Tris pH 8, 150 mM NaCl, 1% (v/v) Nonidet P-40, 0.5% (w/v) sodium deoxycholate, 0.1% (v/v) sodium dodecyl sulfate (SDS)]. To isolate Lewis A bearing glycoproteins, we used the Lewis A-specific JIM84 antibody (Horsley et al., 1993; Fitchette et al., 1999). To capture the IgM antibody, biotinylated anti-rat IgM antibody (Sigma-Aldrich) was incubated with streptavidin-agarose beads (Sigma-Aldrich) in the presence of RIPA dilution buffer (50 mM Tris pH 7.5, 150 mM NaCl, 5 mM EDTA). After binding of anti-rat IgM antibody to the resin, it was washed and incubated with JIM84 antibody. The resin with the two bound antibodies was then mixed with the total protein extracts. After incubation, the resin was washed three times with RIPA dilution buffer and transferred to a Micro Bio-Spin column (Bio-Rad). Bound proteins were subjected to an on-bead digest (Chrestensen et al., 2004) with slight modifications. Briefly, 10 mM dithiothreitol (DTT) in 100 mM NH_4_HCO_3_ buffer was pipetted to the agarose beads and incubated at 60°C for 15 min. 18 mM iodoacetamide in 100 mM NH_4_HCO_3_-buffer was added for alkylation and incubated for 30 min in the dark. Afterwards, trypsin was added (Sequencing Grade modified trypsin, Promega) to a final concentration of 3.5 ng/μL, incubated overnight at 37°C and peptide fragments were isolated on the next day via centrifugation. The supernatant was subjected to a final clean-up step using a C18 Hypersep cartridge (Thermo Fisher Scientific). The cartridge was washed three times with 65% (v/v) acetonitrile in 80 mM ammonium formate buffer pH 3, equilibrated in the same buffer and loaded with the eluted peptides in 100 mM NH_4_HCO_3_ buffer pH 7.8. After washing three times, peptides were eluted with 65% acetonitrile in pH 3 buffer and vacuum dried.

### Identification of Lewis A Bearing Glycoproteins by Mass Spectrometry

Analysis of isolated Lewis A bearing glycoproteins was conducted using nano-LC-ESI-MS as previously described (Zámocký et al., 2020). In short, the peptide fragments were analyzed using a Dionex Ultimate 3000 system interfaced to a maXis 4G ETD QTOF system (Bruker). A Thermo Acclaim PepMap precolumn was coupled to a Thermo Acclaim PepMap 300 RSLC C18 separation column (2 μm particle size, 150 x 0.075 mm) for separation of peptides, running a gradient of 95% solvent A to 32% solvent B over the course of 60 min (solvent A: 0.1% formic acid in HQ-water, solvent B: 0.1% formic acid in acetonitrile), followed by a gradient from 32 to 70% solvent B for 10 min at 0.3 μL/min flow rate. CaptiveSpray nano Booster was used as ion source in positive ion mode. MS spectra were recorded in a range of 150–2200 m/z, and the six highest peaks selected for fragmentation in data dependent acquisition mode. For identification of proteins, the analysis files were converted to XML using Data Analysis 4.0 (Bruker) to allow MS/MS ion searches using MASCOT (embedded in Protein Scape 3.0, Bruker). For protein searches from *N. benthamiana*, a recently published database was used (Schiavinato et al., 2019). For Arabidopsis, the UniProt non-redundant *A. thaliana* database and for rice the non-redundant UniProt *O. sativa japonica* database were used. Proteins identified by at least two peptides and a protein score higher than 80 were accepted. For comparing the MS/MS data to the target sequence via X! Tandem (https://thegpm.org/TANDEM/), the following settings were used: reversed sequences no; check parent ions for charges 1, 2, and 3 yes; models found with peptide log e lower −1 and proteins log e lower −1; residue modifications: oxidation M, W, and deamidation N, Q; isotope error was considered; fragment type was set to monoisotopic; refinement was used with standard parameters; fragment mass error of 0.1 Da and ±7 ppm parent mass error; fragment types b and y ions; maximum parent ion charge of three; missed cleavage sites allowed was set to two; semi-cleavage yes.

### Vector Construction

For expression of identified proteins in *A. thaliana* as well as in leaves of *N. benthamiana*, a modified version of the binary vector pPT2 (Strasser et al., 2005) was used. For expression of KORRIGAN in leaves of *N. benthamiana*, p29-Fc-KOR1 was used (Liebminger et al., 2013). For expression of COBL4, the coding sequence without endogenous signal peptide (bases 1-60) was amplified from cDNA of *A. thaliana* using primers At5g15630_1F (5′-TATATCTAGATATGATCCATTAGATCCTAGTGGTA-3′) and At5g15630_2R (5′-TATAAGATCTTCACCATATTGAGATGAATAGGAGA-3′), digested with XbaI/BglII and ligated into XbaI/BamHI-digested p117 (Shin et al., 2018). g6145 was amplified without the sequence encoding the signal peptide (bases 1–72) from cDNA of *N. benthamiana* using primers g6145_F1 (5′-TATATCTAGAGGAGATCCATTTAAGTTTTTTAACTT-3′) and g6145_R2(5′-TATAGGATCCCTAATAGAACACAGAAAAGATTGCA-3′), digested with XbaI/BamHI and ligated into XbaI/BamHI-digested p117. For expression of CEBIP, a codon-optimized version for *N. benthamiana* lacking the endogenous signal peptide coding sequence (bases 1–84) was ordered from GeneArt (Thermo Fisher Scientific). The DNA sequence was amplified using primers STRINGS_9F (5′-CTTCCGGCTCGTTTGTCTAGA-3′) and STRINGS_2R (5′-AAAAACCCTGGCGGGATCC-3′), digested with XbaI/BamHI and ligated into XbaI/BamHI-digested p117.

### Recombinant Expression and Purification of Glycoproteins

For recombinant expression of identified proteins in *N. benthamiana*, syringe-mediated leaf infiltration of 5-week-old plants was used. *Agrobacterium tumefaciens* strain UIA143 (Strasser et al., 2005) carrying the respective plasmid for expression was grown over night at 29°C and adjusted to an OD_600_ of 0.15 on the next day in infiltration buffer (28 mM glucose, 50 mM 2-(4-morpholino)-ethanesulphonic acid (MES), 2 mM Na_3_PO_4_.12H_2_O, 0.1 mM acetosyringone). p29-Fc-KOR1 was additionally co-infiltrated with silencing suppressor p19 (Garabagi et al., 2012) at an OD_600_ of 0.1. Leaf material was collected 2 days post infiltration (dpi) and snap-frozen. For stable expression in *A. thaliana*, plants were transformed using the floral dip method (Clough and Bent, 1998). Stems of homozygous plants were collected ~5 weeks after sowing. Plant material was disrupted mechanically, and total proteins extracted using RIPA buffer. Recombinantly produced glycoproteins were purified via their fused tags, either GFP for KORRIGAN (GFP-Trap agarose, Chromotek) or mRFP for the other constructs (RFP-Trap agarose, Chromotek). After capture, proteins were eluted in 1.5x Laemmli buffer.

### Deglycosylation and Immunoblotting

Purified proteins from *N. benthamiana* and Arabidopsis Col-0 plants were subjected to deglycosylation with Endoglycosidase H (Endo H, New England Biolabs) according to the manufacturer's instructions. For deglycosylation experiments with Peptide-N-glycosidase F (PNGaseF, New England Biolabs), proteins purified from ΔXT/FT-*N. benthamiana* (Strasser et al., 2008) and *A. thaliana fut11 fut12* (Strasser et al., 2004) were used. For immunoblotting, purified glycoproteins were separated on a 10% (v/v) polyacrylamide gel and transferred to a nitrocellulose membrane. Primary antibodies against GFP and RFP (both purchased from Chromotek) were diluted (1:2000) in PBS + 0.1% (v/v) Tween (PBST) + 1,5% (w/v) BSA, as secondary antibody anti-mouse IgG (Sigma-Aldrich) diluted (1:10.000) in PBST + 1% BSA was used. JIM84 was diluted (1:400) in PBST and anti-rat IgM antibody (Sigma-Aldrich) was used as secondary antibody. Detection via ECL substrate (Super Signal West PICO Plus Chemiluminescent substrate, Thermo Fisher Scientific) was monitored on a Fusion instrument (Vilber).

### Site Specific N-glycan Analysis via Mass Spectrometry

For site-specific N-glycan analysis, purified proteins from wild-type *N. benthamiana* and *A. thaliana* Col-0 were separated on a 10% polyacrylamide gel via SDS-PAGE, stained with Coomassie Brilliant Blue and excised from the gel. An in-gel digest was conducted as previously described (Kolarich and Altmann, 2000) with the exception, that a double-digest of trypsin and chymotrypsin was used for proteolysis.

### Analysis of Cell Wall Constituents

Cell wall constituents were quantified as previously described (Corneillie et al., 2019) with slight modifications. Stems from fully grown Arabidopsis plants were collected after drying, the bottom-most 2 cm removed, and the next 15 cm used for analysis. Alcohol soluble residues were removed by sequential extraction in H_2_O (30 min, 98°C), ethanol (30 min, 76°C), chloroform (30 min, 59°C), and acetone (30 min, 54°C). Remaining cell wall residues were weighed, and percentage of full stem weight calculated. For quantification of cellulose, half of the cell wall residue fraction was incubated in trifluoroacetic acid (TFA) for 3 h with shaking in-between to remove hemicellulose. After washing with acetone, the remaining pellet was dried and weighed. Updegraff-reagent (Updegraff, 1969) was added to the pellet and heated for 30 min at 100°C, washed once with H_2_O and three times with acetone. After air drying overnight, the remaining crystalline cellulose was dissolved in 72% (v/v) sulphuric acid, incubated, H_2_O added and centrifuged. The supernatant was diluted 1:10, mixed with anthrone reagent in sulphuric acid in a 96 well plate, heated to 80°C for 30 min and subsequently absorption was measured at 595 nm using a plate reader (Tecan, Infinite 200 Pro). Cellulose content in the samples was calculated based on a glucose–standard curve and percentage of cellulose per cell wall residues calculated. For determination of lignin, the second half of the cell wall residue pellet was mixed with 25% (v/v) acetyl-bromide solution in acetic acid, heated for 3 h at 50°C and centrifuged. The supernatant was diluted 1:20 in a solution containing 0.4 M NaOH and 18.75 mM hydroxylamine hydrochloride in acetic acid and absorption measured at 280 nm (Nanodrop, Thermo Fisher Scientific). Lignin content was then calculated as percentage of cell wall residues.

### Root Growth Assays

Measurement of root growth dynamics and root growth under stress conditions was conducted as described (Dubiel et al., 2020). In short, seeds of *A. thaliana* Col-0 and mutant plants were sterilized, stratified for 3 days, and grown on half-strength MS medium. For analysis of root growth dynamics, root length was measured after 48, 96, 144, 192, and 240 h. For analysis of root growth under stress conditions, seedlings were transferred to either new plates (mock control), plates containing 150 mM NaCl (salt stress) or plates containing 12% (w/v) PEG6000 corresponding to a water potential of −0.5 MPa for simulation of drought stress via the PEG infusion method (Van Der Weele et al., 2000). After 10 days, plates were scanned, number of lateral root hairs counted, and root length measured using Image J (Schindelin et al., 2012).

## Results

### Identification of Lewis A Bearing Glycoproteins

In this study, we set out to identify glycoproteins harboring the Lewis A trisaccharide on complex N-glycans in various organs of selected plants. To this end, we analyzed total protein extracts from siliques and stems of the dicot model plant *A. thaliana*, shoots of the monocot *O. sativa*, and leaves and stems from *N. benthamiana*, which is frequently used as a production platform for recombinant glycoproteins (Stoger et al., 2014). We isolated Lewis A bearing glycoproteins via the JIM84 antibody (Fitchette et al., 1999), conducted a tryptic digest and subsequently analyzed the resulting peptide fragments via peptide mapping using nano-LC-ESI-MS. To discriminate false positives from actual Lewis A bearing glycoproteins we included a negative control, for which we repeated the same isolation procedure in the absence of the JIM84 antibody. Table 1 lists glycoproteins from *A. thaliana* and *O. sativa* that were identified at least twice in three replicates and were not detected in the negative control. We identified 10 proteins from *A. thaliana*, four of which we detected in both stems and siliques, namely Laccase 4, COBRA-like protein 4 (COBL4), Endoglucanase 25 (also known as KORRIGAN) and Protein trichome-birefringence like 3. All these proteins have numerous potential N-glycosylation sites and a predicted localization in the secretory pathway. Interestingly, many of the identified proteins have a proposed function in secondary cell wall biosynthesis. In addition, we detected Laccase 17, Probable inactive purple-acid-phosphatase and Protein trichome-birefringence-like 33 in siliques as well as O-fucosyltransferase family protein SUB1 in stems of *A. thaliana*. The list obtained from *O. sativa* comprises proteins of various functions. We detected two defense related proteins, Chitin elicitor-binding protein (CEBIP) and Germin-like protein 2–4. Furthermore, Dirigent protein, which is involved in biosynthesis of secondary metabolites, Os02g0615800, a receptor kinase, Beta-Ig-H3 domain-containing protein, a cell surface adhesion protein, and Os08g0503200, a glycerophosphodiester phosphodiesterase. In *N. benthamiana* (Supplementary Table 1) we detected homologs of Endoglucanase 25 as well as a homolog of a COBRA-like extracellular GPI-anchored protein. In addition, we detected homologs of glucosyl hydrolases as well as proteins involved in directional growth and cell expansion such as a mono-copper oxidase-like SKU5 protein (g6145).

**Table 1:** Lewis A bearing glycoproteins from stems and siliques of *A. thaliana* Col-0 and shoots of *O. sativa japonica*.

**UniProt Accession**	**Name**	**Potential N-glycosylation sites**	**Predicted Localization**	**Proposed Function**
***A. thaliana*** **stems**				
O80434	Laccase 4 (IRX12)	14	Secreted	Lignin biosynthesis
Q9LFW3	COBRA-like protein 4 (COBL4)	8	Secreted (GPI-anchored)	Cellulose biosynthesis
Q38890	Endoglucanase 25 (KORRIGAN)	8	Plasma membrane	Cellulose biosynthesis
Q8LED3	Protein trichome birefringence-like 3 (TBL3)	6	Golgi apparatus	Deposition of cellulose
Q9LE45	O-fucosyltransferase family protein (SUB1)	11	Golgi apparatus	Transfer of glycosyl-residues
***A. thaliana*** **siliques**				
Q38890	Endoglucanase 25 (KORRIGAN)	8	Plasma membrane	Cellulose biosynthesis
O80434	Laccase 4 (IRX12)	14	Secreted	Lignin biosynthesis
Q9FJD5	Laccase 17 (LAC17)	15	Secreted	Lignin biosynthesis
Q9LFW3	COBRA-like protein 4 (COBL4)	8	Secreted (GPI-anchored)	Cellulose biosynthesis
Q9LMG7	Probable inactive purple acid phosphatase 2 (PAP2)	8	Secreted	Protein of unknown function
Q8LED3	Protein trichome birefringence-like 3 (TBL3)	6	Golgi apparatus	Secondary cell wall biosynthesis
F4IH21	Protein trichome birefringence-like 33 (TBL33)	6	Golgi apparatus	Secondary cell wall biosynthesis
***O. sativa*** **shoots**				
Q306J3	Dirigent protein (JAC1)	5	Secreted	Biosynthesis of lignans, flavonolignans, and alkaloids
Q8H8C7	Chitin elicitor-binding protein (CEBIP)	11	Plasma membrane	Chitin binding
Q6K7X0	Os02g0615800 protein	21	Plasma membrane	Receptor kinase
Q6F3A5	Beta-Ig-H3 domain-containing protein	2	Secreted	Cell surface adhesion protein
Q6ZFH9	Glycerophosphodiester Phospodiesterase	12	Secreted	Lipid metabolism
Q6ESF0	Germin-like protein 2-4	2	Secreted	Plant defense

*Proteins were affinity-purified using the JIM84 antibody and identified via mass spectrometry using peptide mapping. The number of potential N-glycosylation sites is based on predictions using the NetNGlyc 1.0 Server (http://www.cbs.dtu.dk/services/NetNGlyc/). The localization and proposed function are based on information from the UniProt-database (https://www.uniprot.org/) and predictions using TargetP (http://www.cbs.dtu.dk/services/TargetP/)*.

### The Identified Proteins Are Modified With Lewis A Structures When Recombinantly Expressed

**Figure 1:**
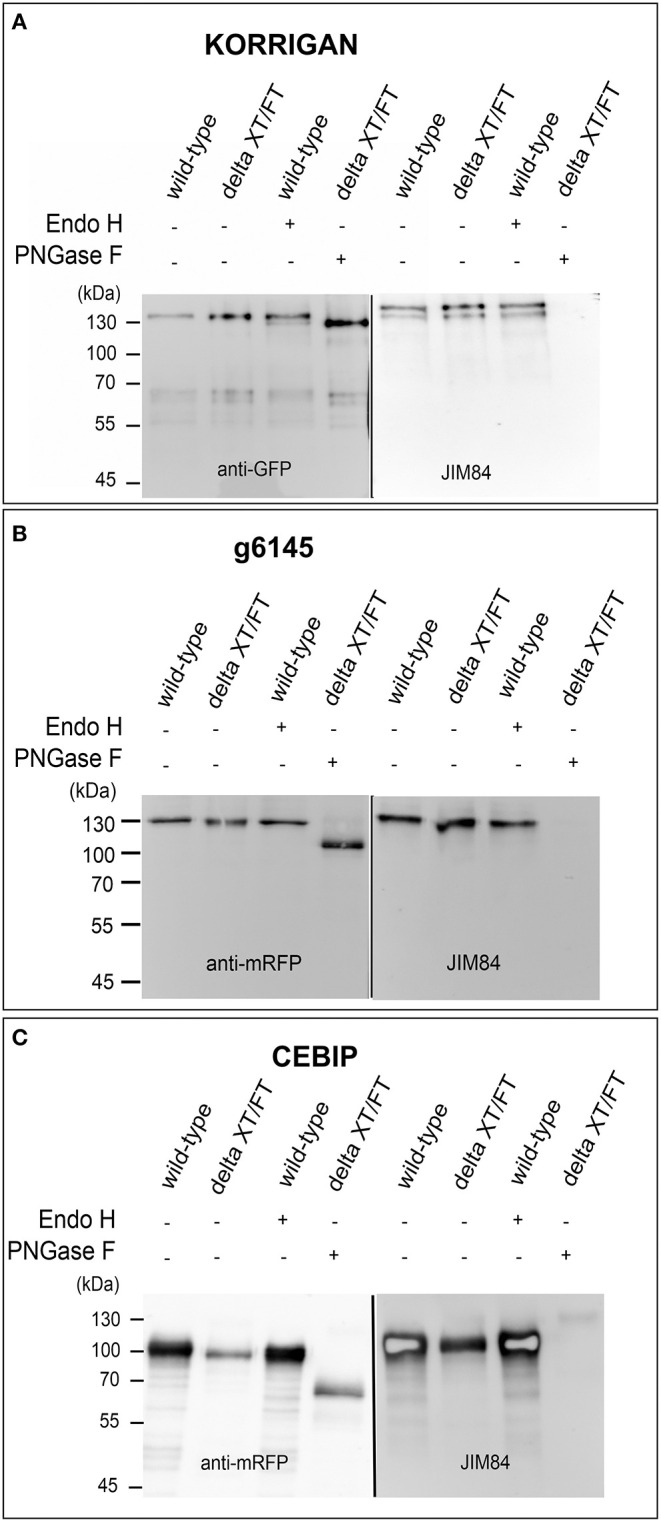
Lewis A bearing complex N-glycans are present on recombinantly produced plant proteins. KORRIGAN **(A)**, g6145 **(B)**, and CEBIP **(C)** were transiently expressed in leaves of wild-type and ΔXT/FT plants of *N. benthamiana* and purified via their fused tag. Expression of proteins was confirmed via immunoblotting using an antibody directed against the fused tag (GFP for KORRIGAN and mRFP for g6145 and CEBIP). Presence of Lewis A was shown using the JIM84 antibody.

**Figure 2:**
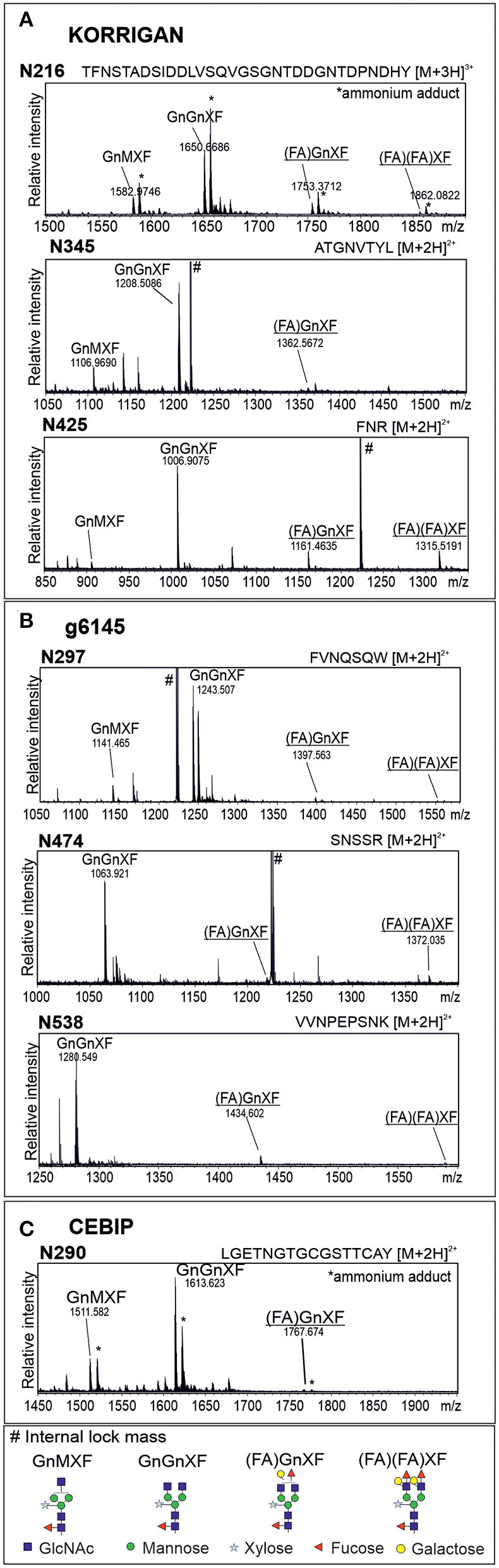
Glycopeptides from recombinantly produced plant proteins display complex N-glycans with Lewis A structures. MS spectra of glycopeptides from KORRIGAN **(A)**, g6145 **(B)**, and CEBIP **(C)**. Proteins were recombinantly expressed in leaves of wild-type *N. benthamiana*, purified via the fused tag and subjected to proteolytic digest using trypsin and chymotrypsin. Nomenclature of N-glycans is according to the ProGlycAn system (http://www.proglycan.com/). Only one N-glycan isoform is indicated. Lewis A bearing glycans are underlined.

We were not able to detect Lewis A bearing glycopeptides on the isolated peptide fractions from the three plant species. Thus, to confirm the presence of Lewis A bearing N-glycans on the identified proteins, we cloned one selected protein from each species into a binary expression vector and expressed it recombinantly as a fusion with a fluorescent protein in leaves of *N. benthamiana*, where, in contrast to *A. thaliana*, Lewis A structures are present (Supplementary Figure 1). After purification, we confirmed the presence of Lewis A both via immunoblotting using the JIM84 antibody and via mass spectrometry using site-specific N-glycan analysis. Figure 1 shows the results of immunoblotting for KORRIGAN from *A. thaliana*, g6145 from *N. benthamiana* and CEBIP from *O. sativa*. A shift is observed upon PNGase F treatment but not upon Endo H treatment, indicating that the majority of N-glycans on these recombinantly expressed glycoproteins are of the complex-type. Furthermore, all proteins show a signal on the JIM84-blot, which is not visible anymore in the PNGase F treated sample indicating that the Lewis A structure is present on N-glycans. Using a combination of trypsin and chymotrypsin for proteolysis, we could detect three peptides with N-glycosylation sites carrying Lewis A on both KORRIGAN and g6145 as well as one site on CEBIP (Figure 2). On all these N-glycosylation sites, Lewis A bearing N-glycans only constitute a minor fraction compared to other structures. We were, however, not able to detect all potential glycopeptides (Supplementary Figure 2), and there might be additional N-glycosylation sites which also carry Lewis A structures.

Next, we compared the N-glycan microheterogeneity of an identified protein expressed either transiently in leaves of *N. benthamiana* or stably in stems of *A. thaliana*. The overall N-glycosylation pattern of COBL4 is very similar between the two plant species. For instance, sites carrying oligomannosidic N-glycans in *N. benthamiana* also carried the same type of N-glycans in *A. thaliana* (Supplementary Figures 3, 4). Lewis A structures were more abundant on glycopeptides carrying N-glycosylation sites N154 and N162 in *N. benthamiana* compared to *A. thaliana*, where Lewis A glycans were hardly detectable. On the other hand, the glycopeptide with site N306 carried a complex N-glycan with Lewis A structures when expressed in *A. thaliana*, which were not found on COBL4 glycopeptides derived from *N. benthamiana*.

### Lewis A-deficient *A. thaliana* Show Normal Stem Morphology and Root Development

**Figure 3:**
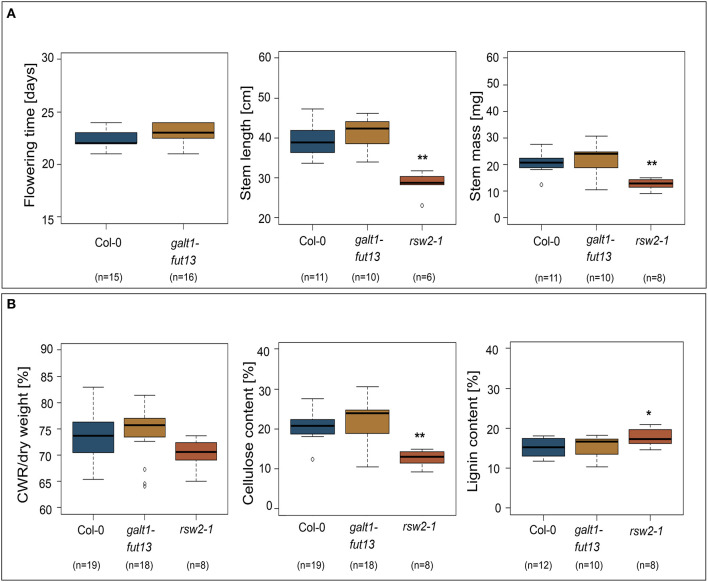
Phenotypic comparison of wild-type and Lewis A-deficient (*galt1 fut13*) plants. *rsw2-1* plants used as controls. The bottom and top of the boxes represent 25 and 75 percent quartiles, respectively, while the horizontal line inside the box indicates the median. The whiskers indicate 1,5x interquartile range (IQR). Asterisks indicate statistically significant differences to Col-0 plants (*p ≤ 0.05, **p ≤ 0.01). **(A)** Comparison of flowering time, length of the plants primary stem, and mass of dried primary stems. **(B)** Content of the primary stems. Cell wall residues (CWR) were determined gravimetrically after sequential removal of alcohol soluble residues. Cellulose and lignin content were determined photometrically as percentage of CWR.

Most proteins identified in both stems and siliques of *A. thaliana* are implicated in biosynthesis of the secondary plant cell wall. We thus hypothesized that plants lacking Lewis A structures on their complex N-glycans might show alterations in stem development or morphology. To test this hypothesis, we made use of a line lacking Lewis A glycans due to T-DNA insertions in genes coding for GALT1 and FUT13 (Supplementary Figure 5). Stems of the *galt1 fut13* double mutant developed normally, exhibiting no differences in flowering time, stem length or mass of dried stems when compared to Col-0 wild-type plants (Figure 3). As a control, we included *rsw2-1* containing a missense mutation in the gene coding for KORRIGAN (Lane et al., 2001), resulting in a dwarfed phenotype and reduced cellulose content. Additionally, we analyzed the content and composition of fully grown dried stems. We analyzed cell wall residues (CWR) gravimetrically after sequential extraction of alcohol soluble residues. Lignin- and cellulose-levels within the fraction of alcohol insoluble residues were measured photometrically using the acetyl bromide method and the anthrone-assay, respectively. We could not find significant differences between *galt1 fut13* and Col-0, indicating that plants develop normal stem morphology in the absence of Lewis A structures.

A frequently observed phenotype in *A. thaliana* lines with mutations along the N-glycosylation pathway are defects in root growth or enhanced susceptibility toward abiotic challenge such as salt stress (Kang et al., 2008; Liebminger et al., 2009). To see whether *galt1 fut13* plants showed alterations in root growth we determined root growth dynamics by measuring root length at 2, 4, 6, 8, and 10 days after sowing (Supplementary Figure 6). The *galt1 fut13* plants showed no difference compared to Col-0. In addition, we analyzed the plants susceptibility to salt and drought stress (Supplementary Figure 6). Again, we could not detect any difference in the *galt1 fut13* line compared to wild-type, indicating normal root development of Lewis A-deficient Arabidopsis plants under abiotic stress conditions.

### Functional *GALT1* and *FUT13* Genes Are Present in Most Natural Accessions of *A. thaliana*

**Figure 4:**
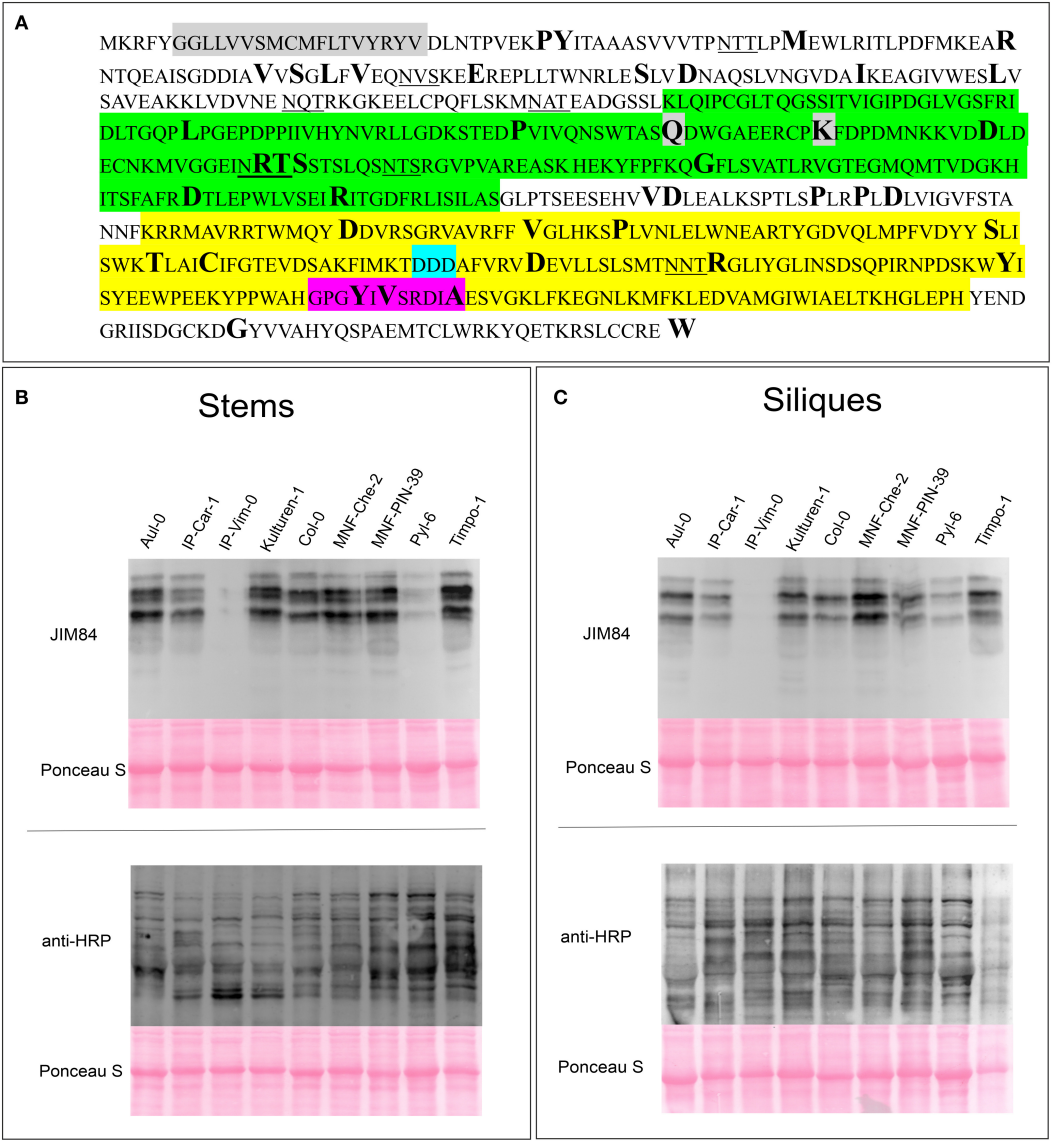
Detection of Lewis A structures in Arabidopsis accessions carrying *GALT1* polymorphisms. **(A)** Protein sequence of GALT1 from Arabidopsis Col-0. The transmembrane domain is shown in gray, the putative galactoside-binding lectin domain in green, and the galactosyltransferase domain in yellow. Additionally, the DXD-motif in the catalytic site is highlighted in light blue and the conserved GxxYxxS as well as the adjacent DxA motif are highlighted in magenta. Underlined amino acids indicate N-glycosylation sequons. Sites depicted in bold show positions of amino acids, for which we could find variations in the 1001 genome database. **(B,C)** Immunoblot analysis of the indicated Arabidopsis accessions. Total protein extracts from stems and siliques, respectively, were analyzed with antibodies directed against Lewis A bearing (JIM84) and plant N-glycans carrying β1,2-xylose and core α1,3-fucose (anti-HRP).

Despite the lack of an obvious phenotype in Lewis A-deficient *A. thaliana* plants, taking into account the strong evolutionary conservation of the carbohydrate epitope we reasoned that the ability to synthesize Lewis A structures might still be an advantage for plants and play a yet unknown role for plant fitness. To examine whether there are Arabidopsis accessions containing SNPs, deletions or insertions in the genes coding for FUT13 and GALT1 we searched the 1001 genomes database using the POLYMORPH 1001 tool (https://tools.1001genomes.org/polymorph/). We found only one SNP with a predicted high impact, an alteration in the nucleotide sequence leading to the formation of a premature stop codon in the *FUT13* gene. This SNP is present in 55 accessions and results in the expression of a truncated FUT13 protein where the two C-terminal amino acids (“GV”) are missing. Since there is some amino acid sequence variation at the C-terminus of FUT13 from different species we consider it unlikely that the truncation completely abolishes FUT13 function. In addition, polymorphisms were detected in the *FUT13* gene which might have an impact on FUT13 activity. As GALT1 catalyzes the rate limiting reaction of Lewis A biosynthesis, we decided to investigate polymorphisms in the *GALT1* gene in more detail. We found only one accession, IP-Orb-10, containing a premature stop codon instead of the codon encoding the last amino acid W643. Figure 4 shows the amino acid sequence of GALT1 and highlights amino acids for which we found polymorphisms in the database. Polymorphisms leading to amino acid changes are found in the putative lectin domain and in the GALT1 catalytic domain. We selected different accessions containing amino acid changes in various regions of GALT1 (Supplementary Table 2) and analyzed the presence of Lewis A in total protein extracts from stems and siliques by immunoblotting (Figures 4B,C; Supplementary Figure 7). While most lines including IP-Orb-10 showed similar Lewis A levels as Col-0, some accessions such as Kulturen-1, MNF-Che-2, and MNF-Pin-39 displayed a slightly stronger signal with JIM84, especially in siliques. One line, PYL-6, showed only very low levels of Lewis A in both stems and siliques, and IP-Vim-0 appeared devoid of detectable Lewis A structures in siliques and displayed only a very faint signal in stems. To confirm the absence of Lewis A in IP-Vim-0 we repeated the JIM84 blot and compared the signal to extracts from Lewis A-deficient plants *galt1-fut13* and *galt1* (Supplementary Figure 8). We observed no JIM84 signal in IP-Vim-0 stems, indicating the absence of Lewis A structures in this line. In PYL-6 and IP-Vim-0 we found that GALT1 and FUT13 transcripts are expressed and we confirmed the polymorphisms in the region coding for the GALT1 catalytic domain in the IP-Vim-0 cDNA by sequencing (Supplementary Figure 9). In contrast to the result for the JIM84 antibody, both accessions show a signal with an antibody directed against N-glycans with β1,2-xylose and core α1,3-fucose (Figures 4B,C) indicating normal processing of oligomannosidic to complex N-glycans. Furthermore, we tested various ecotypes of *A. thaliana* and were able to detect Lewis A structures in all of them (Supplementary Figure 10). In summary, the data suggest that SNPs occurring in conserved regions of GALT1 are rare exceptions in natural accessions of *A. thaliana*.

## Discussion

The Lewis A trisaccharide on complex plant N-glycans is an evolutionarily conserved modification throughout the plant kingdom (Fitchette et al., 1999; Wilson et al., 2001; Léonard et al., 2004; Strasser et al., 2007). Here, we report the isolation of 10 proteins from *A. thaliana*, seven proteins from *O. sativa* and nine proteins from *N. benthamiana* modified with Lewis A. The relatively low number of identified Lewis A bearing glycoproteins can have two possible explanations: (1) either only a low number of specific glycoproteins are modified with Lewis A structures or (2) Lewis A structures are present on various glycoproteins in the *trans*-Golgi, the plasma membrane and the apoplast, but occur in so small quantities that we could identify only the most abundant glycoproteins or the ones carrying complex N-glycans with high amounts of Lewis A, respectively. Based on these possibilities we cannot rule out that we only enriched a certain population of Lewis A bearing glycoproteins using the described purification approach. Overall, the information about Lewis A bearing glycoproteins published in literature is rather scarce. In a recent study investigating N-glycan microheterogeneity in, among others, inflorescence stems of *A. thaliana* (Zeng et al., 2018), the authors report the detection of biantennary complex N-glycans with Lewis A structures on three glycoproteins, among them Laccase 4 (At2g38080.1), which was also identified in our approach in both Arabidopsis stems and siliques. Furthermore, an uncharacterized potentially GPI-anchored protein (At3g06035) was detected. This protein was also found once in one of our experiments but was not included in our list which contains only proteins detected at least twice in three experiments. The third protein reported by the authors, DUF2921 (At1g52780), was not identified in our experiments. In another publication (Zhang et al., 2011), the authors were able to detect monoantennary Lewis A on one protein, homologous to blue copper binding protein (At4g12880). No data has so far been published about Lewis A-bearing glycans on endogenous proteins from *N. benthamiana*. However, Lewis A was reported to be present on recombinantly produced glycoproteins such as human erythropoietin (Castilho et al., 2013). In *O. sativa*, which carries considerable amounts of N-glycans with Lewis A modifications (Léonard et al., 2004), monoantennary Lewis A was detected on nucleotide pyrophosphatase/phosphodiesterases when overexpressed in rice cell culture (Kaneko et al., 2016). These proteins were not identified in our proteomics approach. While the proteins identified in rice show no clear pattern with regard to their proposed functions, the proteins identified in *A. thaliana*, and to a lesser extent also in *N. benthamiana*, show a clear bias toward cell wall biosynthesis. However, the Lewis A-deficient *galt1 fut13* line showed no differences in respect to mass and length of the primary stem, flowering time or constituents of the secondary cell wall, such as cell wall residues, cellulose, and lignin. Furthermore, Lewis A-deficient plants showed normal root development, also under abiotic stress. This is in agreement with previous reports (Strasser et al., 2007; Rips et al., 2014; Basu et al., 2015) which described the lack of an observable phenotype. Furthermore, Basu and colleagues showed that GALT1 is the only member of the CAZy GT31-family not involved in hydroxyproline O-galactosylation of arabinogalactan-proteins (AGPs). While T-DNA insertion lines of other GALTs showed abnormalities of root hairs, seed coat mucilage, silique morphology or pollen tube development, no such phenotype was reported for GALT1-deficient plants (Basu et al., 2015). Interestingly though, FUT13 and GALT1 appear to be strongly conserved among the natural accessions found in the 1001 genome project. Out of 16 selected lines, only one line, IP-Vim-0, lacks detectable levels of Lewis A containing N-glycans in extracts from stems and siliques. This line has two polymorphisms in the *FUT13* gene that alter the FUT13 protein (T294K and 399 instead of 401 amino acids) and may also affect the FUT13 activity. While the truncated FUT13 sequence is found in 55 different accessions, the T294K polymorphism is only found in one other line IP-Vis-0. In the *GALT1* gene from IP-Vim-0 four polymorphisms are present that lead to changes of the amino acid sequence. Two of the changes (Q243L and K253N) are also found in Lewis A-positive lines such as MNF-Pin-39 and therefore do not inactivate GALT1. The other two polymorphisms are present in a conserved sequence motif that is present in the catalytic domain of CAZy GT31 family members (Qu et al., 2008; Petit et al., 2020). The conserved GxxYxxS motif (also called motif IV in the GT31 family members) constitutes the C-terminal part of a flexible G-loop. The loop is involved in donor substrate binding and is close to the active site of the enzyme. A DxA motif that is highly conserved in plant GT31 sequences (Qu et al., 2008) is directly following the GxxYxxS motif. While in Col-0 this sequence stretch is **G**PG**Y**IV**S**R**D**I**A**, it is **G**PG**N**IV**S**R**D**I**E** in IP-Vim-0. Both mutations very likely abolish the enzymatic activity of GALT1 and lead to the absence of Lewis A bearing N-glycans in IP-Vim-0. This line has been defined as a relict, meaning high genomic divergence from geographically proximal accessions (Alonso-Blanco et al., 2016) and may represent a rare exception of a plant line with an impaired pathway for the biosynthesis of Lewis A structures on complex N-glycans. Although we cannot rule out that other amino acid substitutions in GALT1 or FUT13 have a similar effect, it seems that the Lewis A trisaccharide structure is still highly conserved in Arabidopsis accessions occurring in different geographical regions. Together with the presence of the modification in all so far analyzed plant species these data hint at the existence of an evolutionary selection pressure to maintain the Lewis A glycan epitope. Despite many efforts, the role of this complex N-glycan modification remains a mystery in plants.

## Data Availability Statement

The original contributions presented in the study are included in the article/Supplementary Material, further inquiries can be directed to the corresponding author.

## Author Contributions

GB, EV, and RS designed the experiments. GB and DM conducted the experiments. GB, DM, EV, FA, and RS analyzed the results. GB and RS wrote the paper. All authors have made a substantial and intellectual contribution to the work and approved it for publication.

## Conflict of Interest

The authors declare that the research was conducted in the absence of any commercial or financial relationships that could be construed as a potential conflict of interest.
