# Supplementary material for: Lewis A Glycans Are Present on Proteins Involved in Cell Wall Biosynthesis and Appear Evolutionarily Conserved Among Natural Arabidopsis thaliana Accessions
Source: Front Plant Sci. 2021 Mar 11;12:630891. doi: 10.3389/fpls.2021.630891 (PMC7991798; doi:10.3389/fpls.2021.630891)
Supplement: Supplementary file 1 [file Data_Sheet_1.PDF]

Supplementary Material

Lewis A glycans are present on proteins involved in cell wall biosynthesis and appear evolutionarily conserved among natural *Arabidopsis thaliana* accessions

Gernot Beihammer, Daniel Maresch, Friedrich Altmann, Els J.M. Van Damme, Richard Strasser

Contents

Table S1. List of proteins identified in leaves and stems of *N. benthamiana*.

Table S2. Accessions of *A. thaliana* from the 1001 genome project for which Lewis A levels were monitored in stems and siliques.

Figure S1. Immunoblot analysis of total protein extracts from leaves of *N. benthamiana*.

Figure S2. Site-specific N-glycan analysis of KORRIGAN, g6145 and CEBIP expressed in leaves of *N. benthamiana*.

Figure S3. Site-specific N-glycan analysis of COBL4 purified from leaves of infiltrated *N. benthamiana*.

Figure S4. Site-specific N-glycan analysis of COBL4 purified from stems of stably expressing Arabidopsis Col-0 plants.

Figure S5. Immunoblot analysis of total protein extracts from stems and siliques of wild-type and Lewis A-deficient Arabidopsis plants, respectively.

Figure S6. Root growth analysis of Col-0 and *galt1-fut13* plants.

Figure S7. Immunoblot analysis of total protein extracts from stems and siliques of various accessions of *A. thaliana* containing amino acid variations in GALT1.

Figure S8. Immunoblot analysis of total protein extracts from stems of Col-0, IP-Vim-0, and the Lewis A-deficient lines *galt1-fut13* and *galt1*

Figure S9. Analysis of *GALT1* and *FUT13* transcripts in stems of Col-0, PYLl-6 and IP-Vim-0.

Figure S10. Immunoblot analysis of total protein extracts from stems and siliques of various ecotypes of *A. thaliana*.

Table S1. List of proteins identified in leaves and stems of *N. benthamiana*. Proteins were affinity-purified using the JIM84 antibody and identified via mass spectrometry using peptide mapping. The number of potential N-glycosylation sites is based on predictions using the NetNGlyc 1.0 Server (http://www.cbs.dtu.dk/services/NetNGlyc/). The localization and proposed function are based on information from the UniProt-database (https://www.uniprot.org/) and predictions using TargetP (http://www.cbs.dtu.dk/services/TargetP/).

N. benthamiana stems				
**Identifier**	**Potential N-glycosylation sites**	**Predicted localization**	**Proposed function**	**Homologue of (protein from *A. thaliana*)**
**g17123.t1**	9	Plasma membrane	Cellulose biosynthesis	Endoglucanase 25
**g6145.t1**	14	Secreted (GPI-anchored)	Involved in directional growth processes	Mono-copper oxidase-like SKU5
**g37143.t1**	11	Golgi apparatus	Involved in coaction of cryptochrome and phytochrome	Calcium ion binding protein
**g59335.t1**	10	Plasma membrane	Cellulose biosynthesis	Endoglucanase 25
**g99754.t1**	9	Plasma membrane	Hydrolysis of glucosidic linkages	Glucan endo-1,3-beta-glucosidase 1
**g54518.t1**	10	Plasma membrane	Hydrolysis of glucosidic linkages	Glucan endo-1,3-beta-glucosidase 1
**g23587.t1**	9	Plasma membrane	Hydrolysis of glucosidic linkages	O-Glycosyl hydrolase family 17 protein
**g8142.t1**	9	Secreted (GPI-anchored)	Direction of cell expansion	COBRA-like extracellular GPI-anchored protein family
N. benthamiana leaves				
**Identifier**	**Potential N-glycosylation sites**	**Predicted localization**	**Proposed function**	**Homologue of (protein from *A. thaliana*)**
**g6145.t1**	14	Secreted (GPI-anchored)	Involved in directional growth processes	Mono-copper oxidase-like SKU5
**g17123.t1**	9	Plasma membrane	Cellulose biosynthesis	Endoglucanase 25
**g23587.t1**	9	Plasma membrane	Hydrolysis of glucosidic linkages	O-Glycosyl hydrolase family 17 protein
**g54518.t1**	10	Plasma membrane	Hydrolysis of glucosidic linkages	Glucan endo-1,3-beta-glucosidase 1
**g21104.t1**	7	Secreted	Hydrolysis of xylose from xyloglucan	Alpha-xylosidase 1

Table S2. Accessions of *A. thaliana* from the 1001 genome project for which Lewis A levels were monitored in stems and siliques. The *GALT1* (At1g26810) polymorphisms relative to the reference genome (https://tools.1001genomes.org/polymorph/) as well as the geographic location of their collection are listed. Only non-silent mutations are included in the list.

Accession ID	Name	Polymorphisms	Resulting amino acid variation	Country
265	PYL-6	833 G>A	R278Q	FRA
1925	MNF-Che-2	1256 A>C	D419A	USA
2016	MNF-Pin-39	728 A>T	Q243L	USA
		759 A>C	K253N	
		1492 G>A	D498N	
4857	UKSW06-257	728 A>T	Q243L	UK
		759 A>C	K253N	
		1421 G>A	C474Y	
6012	Eden-7	759 A>C	K253N	SWE
		1843 G>C	G612R	
7107	Durh-1	728 A>T	Q243L	UK
		759 A>C	K253N	
		1421 G>A	C474Y	
7520	Lp2-2	1052 G>A	R351Q	CZE
		759 A>C	K253N	
8240	Kulturen-1	619 C>T	L207P	SWE
		620 T>C		
		728 A>T	Q243L	
		759 A>C	K253N	
8249	Vimmerby	728 A>T	Q243L	SWE
		759 A>C	K253N	
		1390 A>T	S464C	
9530	IP-Car-1	355 A>T	I119F	ESP
		728 A>T	Q243L	
		759 A>C	K253N	
		1126 G>T	V376F	
9565	IP-Orb-10	1256 A>C	D419A	ESP
		1928 G>A	W643stop	
9598	IP-Vim-0	728 A>T	Q243L	ESP
		759 A>C	K253N	
		1657 T>A	Y553N	
		1679 C>A	A560E	
9647	Basen-1	728 A>T	Q243L	ITA
		759 A>C	K253N	
		1162 C>T	P388S	
9701	Ivano-1	759 A>C	K253N	BUL
		1171 C>T	P391S	
9822	Aul-0	1256 A>C	D419A	ESP
9968	Timpo-1	239 C>T	S80L	ITA

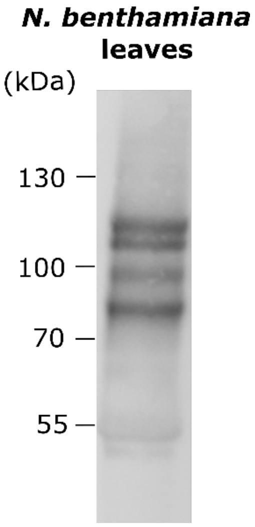

Figure S1. Immunoblot analysis of total protein extracts from leaves of *N. benthamiana*. Proteins were extracted using RIPA buffer, separated via SDS-PAGE and Lewis A bearing glycoproteins detected using the JIM84 antibody.

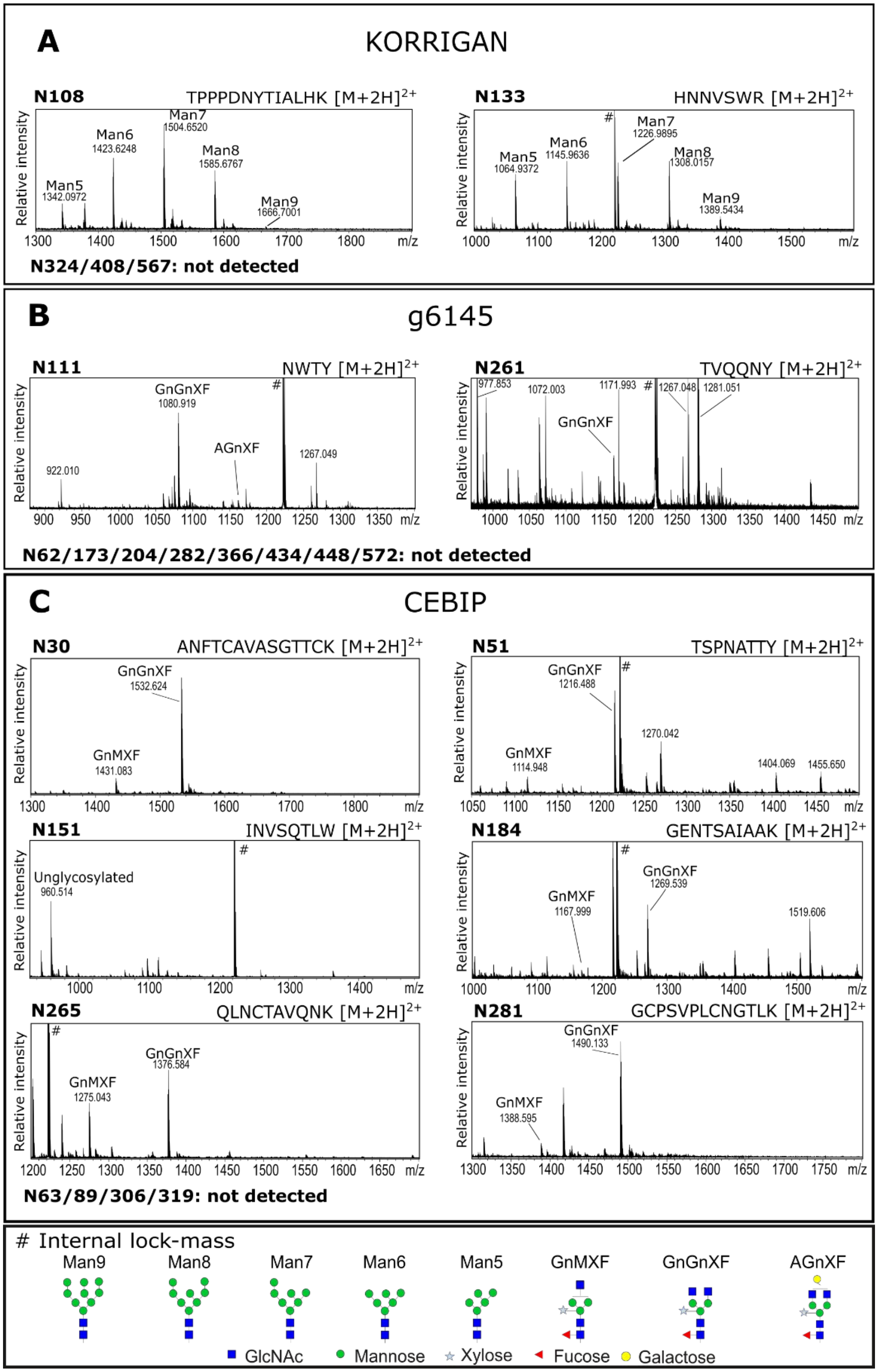

Figure S2. Site-specific N-glycan analysis of KORRIGAN, g6145 and CEBIP expressed in leaves of *N. benthamiana*. The spectra show sites where Lewis A structures were not detected. Proteins were

purified via their fused tag, separated via SDS-PAGE, excised from the gel and subjected to trypsin/chymotrypsin digestion and MS-analysis.

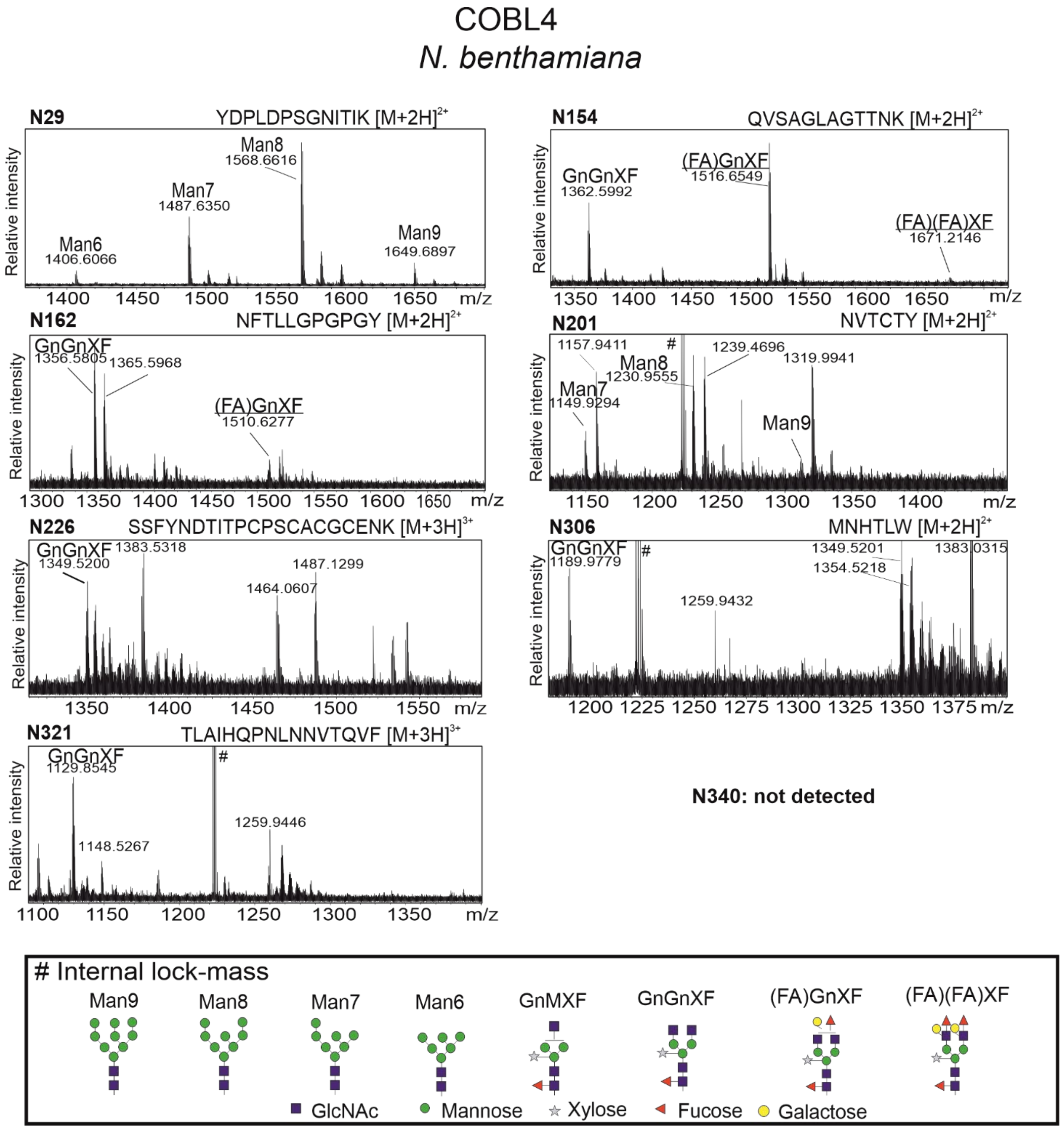

Figure S3. Site-specific N-glycan analysis of COBL4 purified from leaves of infiltrated *N. benthamiana*. Proteins were purified, separated via SDS-PAGE, excised from the gel and subjected to a trypsin/chymotrypsin digestion and MS-analysis. N-glycans decorated with Lewis A structures are underlined.

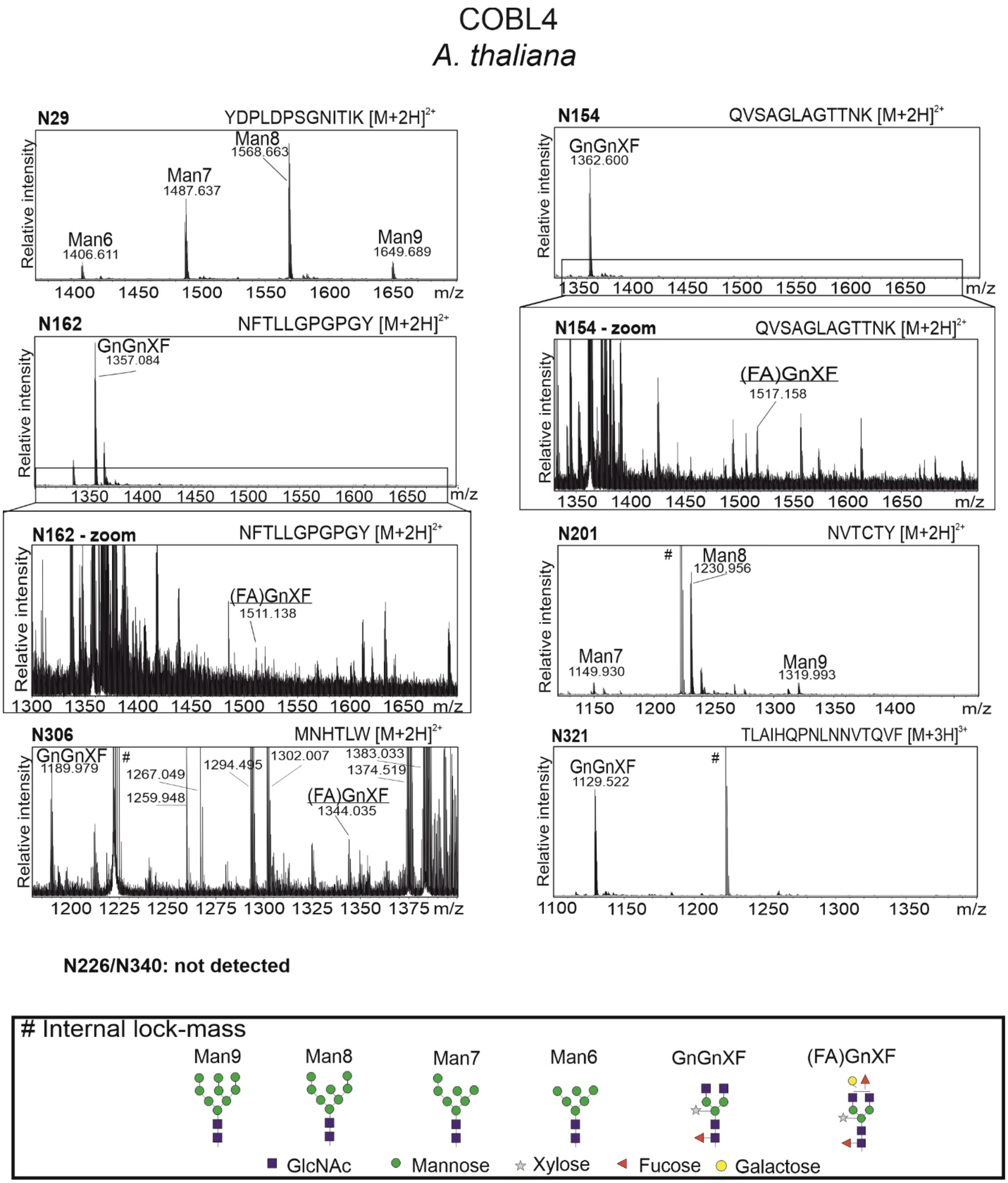

Figure S4. Site-specific N-glycan analysis of COBL4 purified from stems of stably expressing Arabidopsis Col-0 plants. Proteins were purified, separated via SDS-PAGE, excised from the gel and

subjected to a trypsin/chymotrypsin digestion and MS-analysis. N-glycans decorated with Lewis A structures are underlined.

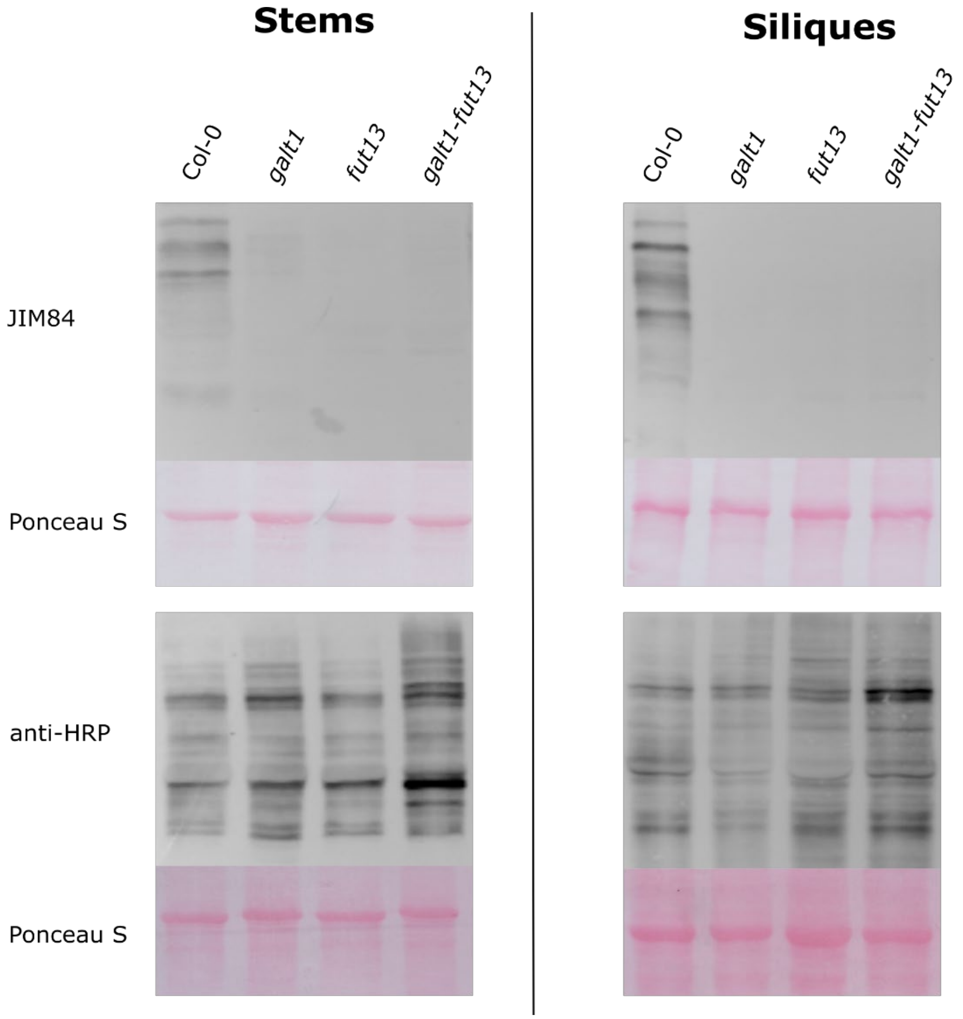

Figure S5. Immunoblot analysis of total protein extracts from stems and siliques of wild-type and Lewis A-deficient Arabidopsis plants, respectively. The JIM84 antibody was used to detect glycoproteins decorated with Lewis A structures, anti-HRP antibody for detection of plant N-glycans carrying β1,2-xylose and core α1,3-fucose.

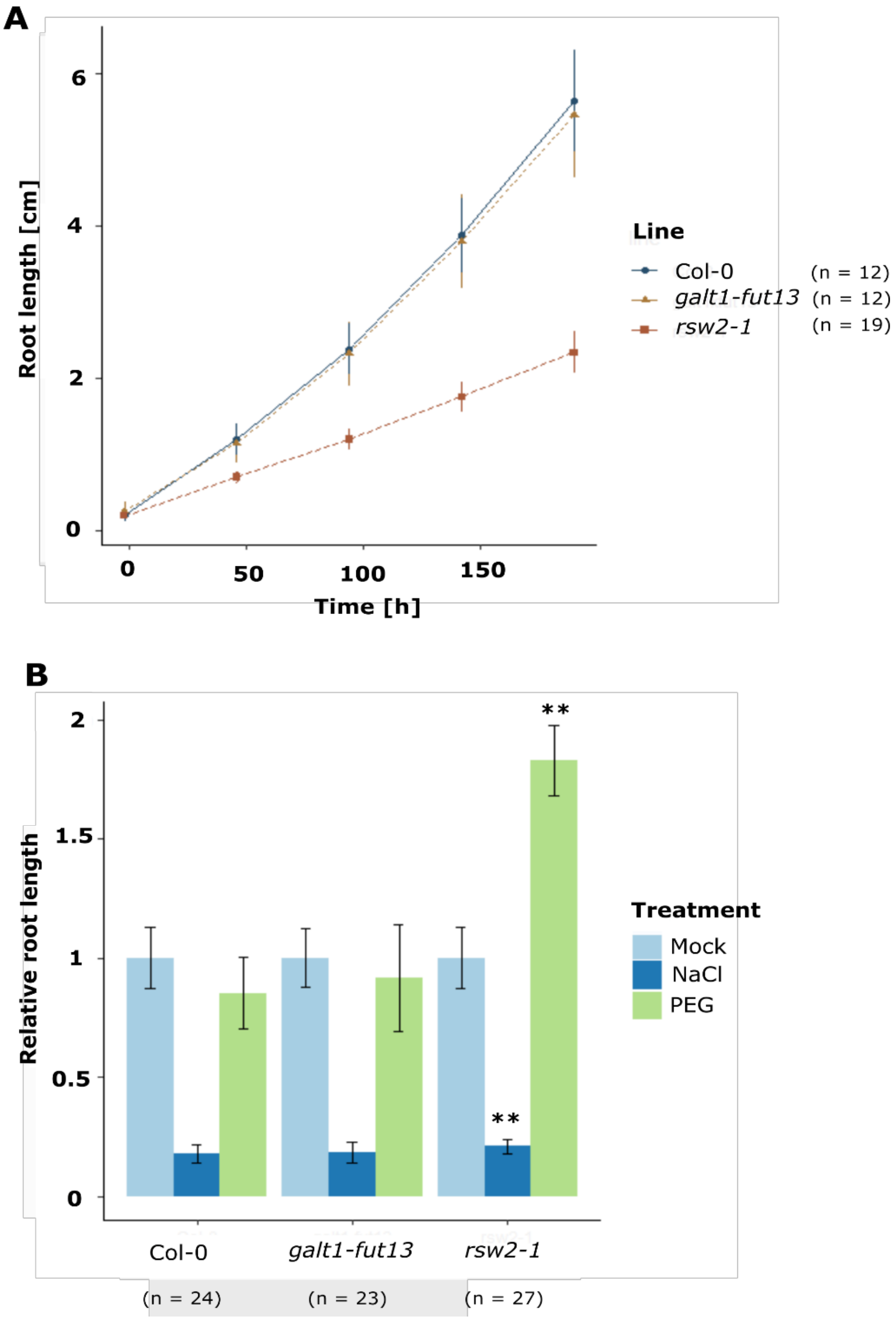

Figure S6. Root growth analysis of Col-0 and *galt1-fut13* plants. *rsw2-1* was included as a control. **(A)** Analysis of root growth dynamics of plants grown on half-strength MS-medium. Following stratification, root length was measured after 48, 96, 144, 192 and 240 hours. Error bars indicate standard deviation. **(B)** Abiotic stress treatment of seedlings using either 150 mM NaCl or PEG6000 at a water potential of -0.5 MPa to mimic drought stress. Error bars represent standard deviation, asterisks indicate statistically significant differences in the relative root lengths compared to Col-0 seedlings based on a Student's t-test (* $p \leq 0.05$, ** $p \leq 0.01$).

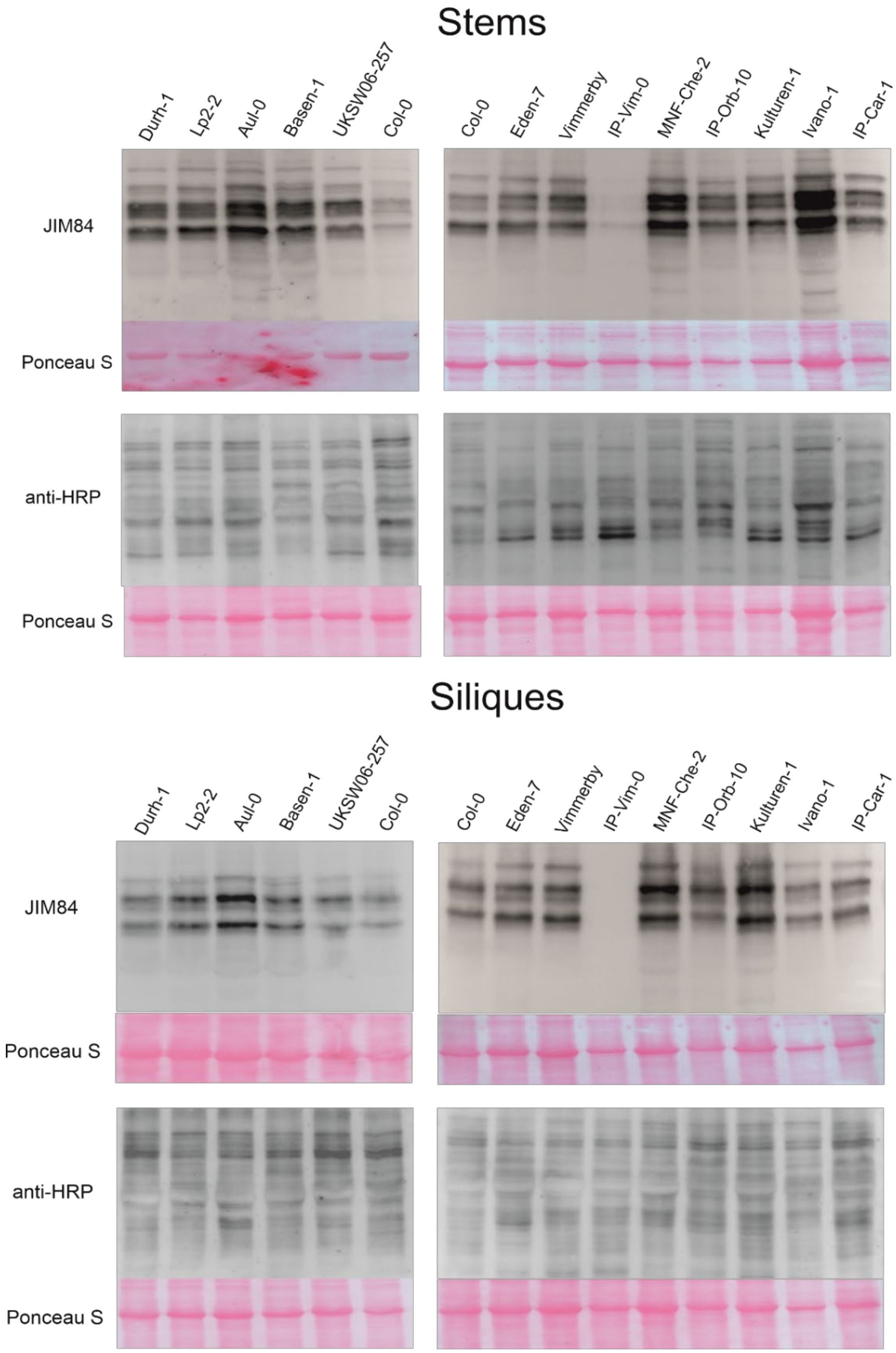

Figure S7. Immunoblot analysis of total protein extracts from stems and siliques of various accessions of *A. thaliana* containing amino acid variations in GALT1. Proteins were extracted using RIPA buffer, separated on an SDS-PAGE and either Lewis A bearing glycoproteins detected using the JIM84

antibody or plant N-glycans carrying β1,2-xylose and core α1,3-fucose detected using the anti-HRP antibody.

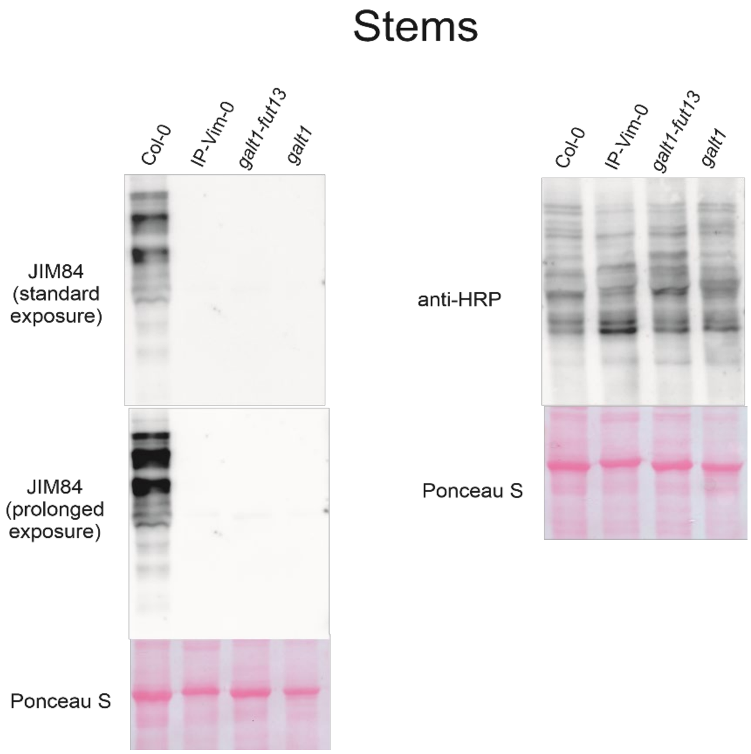

Figure S8. Immunoblot analysis of total protein extracts from stems of Col-0, IP-Vim-0 and Lewis A-deficient lines of *A. thaliana*. Proteins were extracted using RIPA buffer, separated on an SDS-PAGE and either Lewis A bearing glycoproteins detected using the JIM84 antibody or plant N-glycans carrying β1,2-xylose and core α1,3-fucose detected using the anti-HRP antibody.

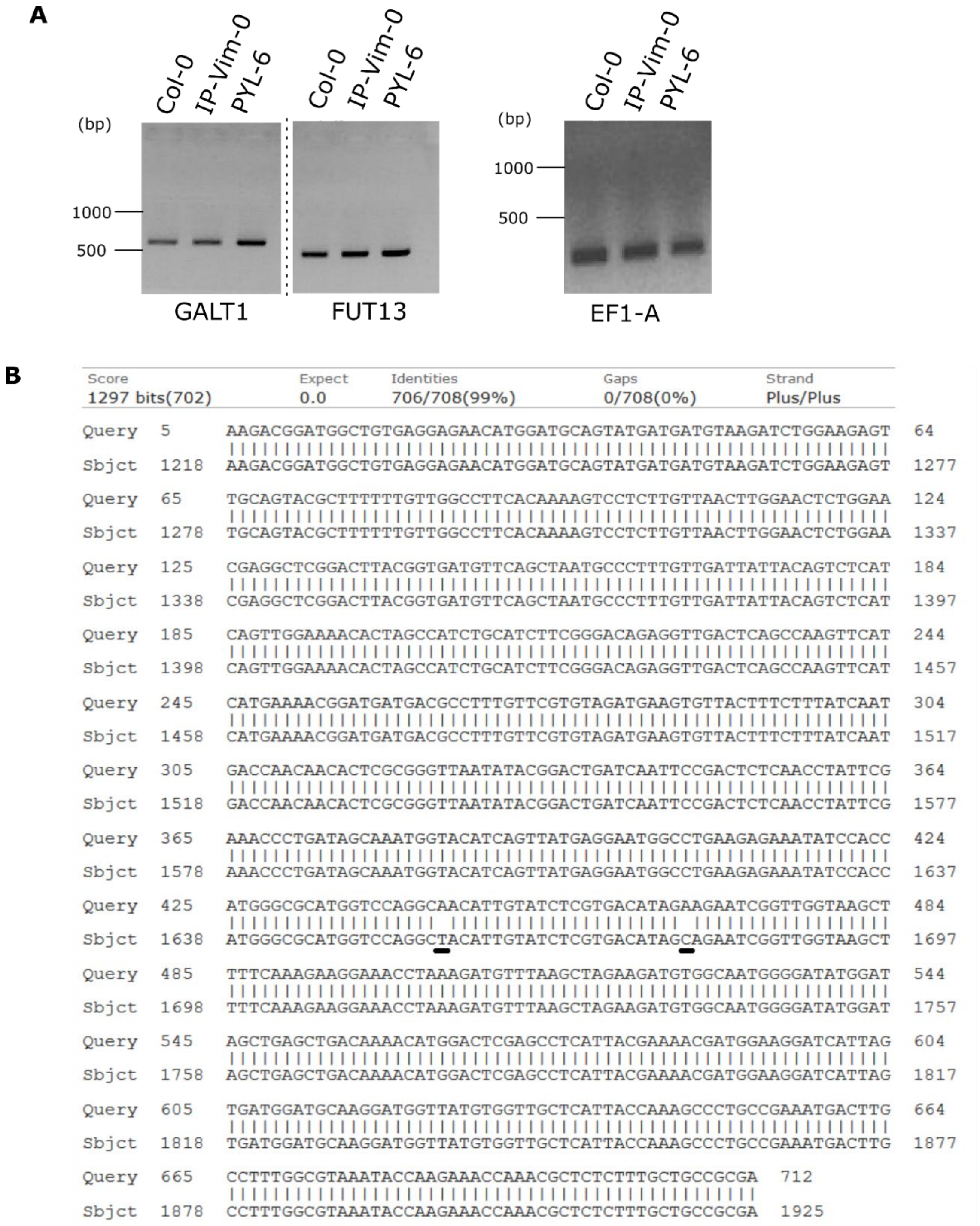

Figure S9. Analysis of *GALT1* and *FUT13* transcripts in stems of Col-0, PYL-6 and IP-Vim-0. **(A)** PCR amplification of *GALT1* and *FUT13* cDNA obtained from RNA extracted from stems of the different ecotypes. *EF1-ALPHA* (EF1-A, At5g60390) was included as a reference gene. **(B)** Alignment of bases 1218-1925 of *GALT1* in Col-0 (lower sequence) and IP-Vim-0 (upper sequence) after sequencing. SNPs are underlined in black. The alignment was done using Blast (https://blast.ncbi.nlm.nih.gov/Blast.cgi?PROGRAM=blastn&PAGE_TYPE=BlastSearch&LINK_LOC=blasthome).

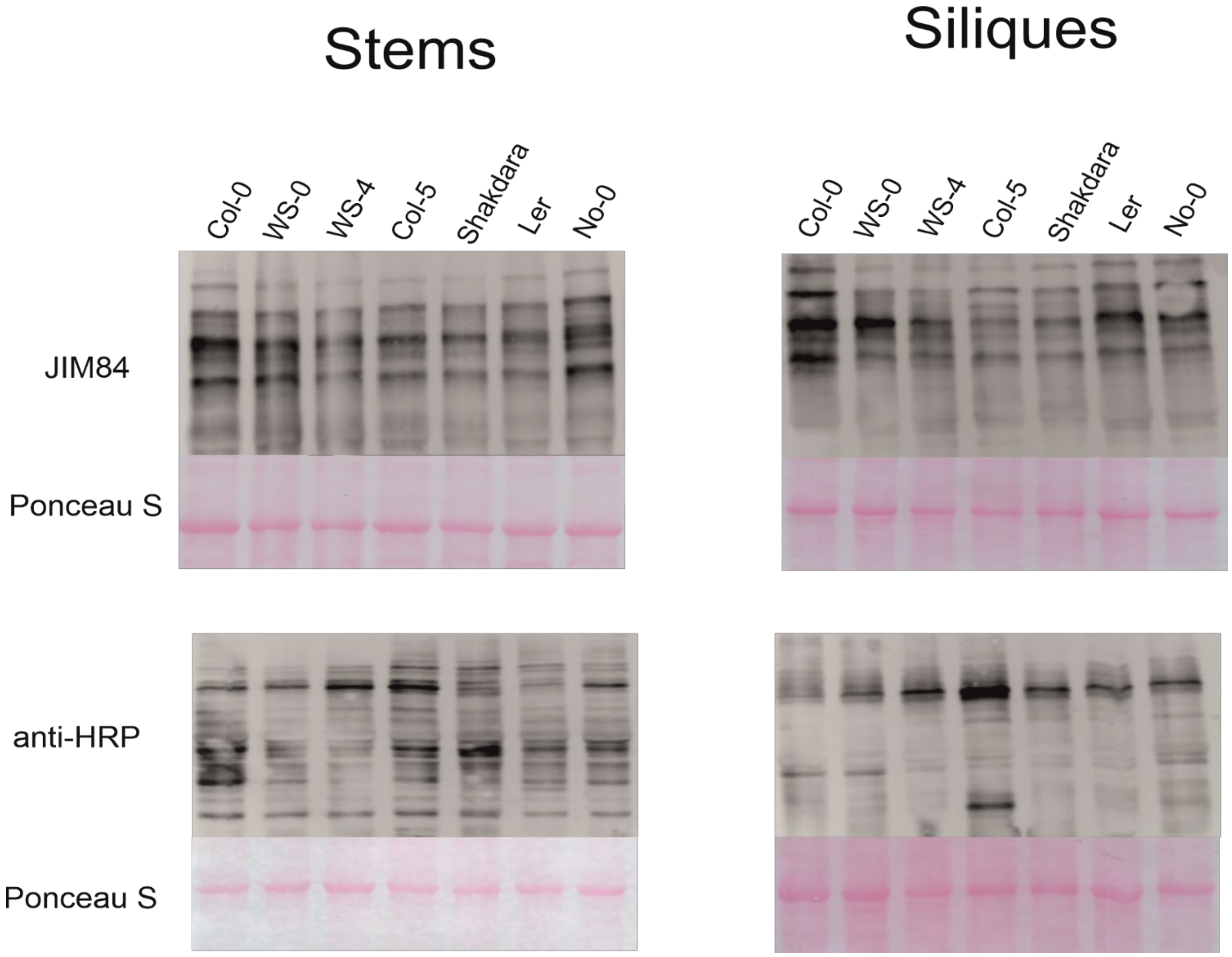

Figure S10. Immunoblot analysis of total protein extracts from stems and siliques of various ecotypes of *A. thaliana*. Proteins were extracted using RIPA buffer, separated on an SDS-PAGE and either Lewis A bearing glycoproteins detected using the JIM84 antibody or plant N-glycans carrying β1,2-xylose and core α1,3-fucose detected using the anti-HRP antibody.
